# What can we learn from more than 1,000 Brazilian patients at risk of hereditary cancer?

**DOI:** 10.3389/fonc.2022.963910

**Published:** 2022-09-05

**Authors:** Ana Carolina Rathsam Leite, Daniele Assad Suzuki, Allan Anderson Lima Pereira, Natalia Polidorio Machado, Romualdo Barroso-Sousa, Tatiana Strava Correa, Fernanda Cesar Moura, Igor Alexandre Protzner Morbeck, Brenda Pires Gumz, Luiza Dib Batista Bugiato Faria, Gustavo dos Santos Fernandes, Renata Lazari Sandoval

**Affiliations:** Department of Oncology, Hospital Sírio-Libanês, Brasília, Distrito Federal, Brazil

**Keywords:** hereditary cancer, cancer predisposition, multigene analyses, genetic testing access, cancer risk assessment

## Abstract

**Background:**

Identifying individuals at a higher risk of developing cancer is a major concern for healthcare providers. Cancer predisposition syndromes are the underlying cause of cancer aggregation and young-onset tumors in many families. Germline genetic testing is underused due to lack of access, but Brazilian germline data associated with cancer predisposition syndromes are needed.

**Methods:**

Medical records of patients referred for genetic counseling at the Oncogenetics Department at the Hospital Sírio-Libanês (Brasília, DF, Brazil) from July 2017 to January 2021 were reviewed. The clinical features and germline findings were described. Detection rates of germline pathogenic/likely pathogenic variant (P/LPV) carriers were compared between international and Brazilian guidelines for genetic testing.

**Results:**

A total of 1,091 individuals from 985 families were included in this study. Most patients (93.5%) had a family history of cancer, including 64% with a family member under 50 with cancer. Sixty-six percent of patients (720/1091) had a personal history of cancer. Young-onset cancers (<50 years old) represented 62% of the patients affected by cancer and 17% had multiple primary cancers. The cohort included patients with 30 different cancer types. Breast cancer was the most prevalent type of cancer (52.6%). Germline testing included multigene panel (89.3%) and family variant testing (8.9%). Approximately 27% (236/879) of the tested patients harbored germline P/LPVs in cancer susceptibility genes. *BRCA2*, *BRCA1*, and *TP53* were the most frequently reported genes, corresponding to 18.6%, 14.4%, and 13.5% of the positive results, respectively. Genetic testing criteria from international guidelines were more effective in identifying carriers than the Brazilian National Agency of Supplementary Health (ANS) criteria (92% vs. 72%, p<0.001). Forty-six percent of the cancer-unaffected patients who harbored a germline P/LPV (45/98) would not be eligible for genetic testing according to ANS because they did not have a family variant previously identified in a cancer-affected relative.

**Conclusion:**

The high detection rate of P/LPVs in the present study is possibly related to the genetic testing approach with multigene panels and cohort’s characteristics, represented mainly by individuals with a personal or family history of young-onset cancer. Testing asymptomatic individuals with suspicious family history may also have contributed to a higher detection rate. A significant number of carriers would not have been identified using ANS criteria for genetic testing.

## Introduction

Carriers of cancer predisposition syndromes (CPSs) are at a higher risk of developing cancer. Familial aggregation, early-onset cancer, and the risk of multiple primary cancers are shared characteristics among CPSs (1, 2). Genetic counseling, modified surveillance, and risk-reduction strategies are essential in these scenarios. Therefore, health professionals involved in comprehensive health care, especially in the diagnosis and treatment of cancer, must be able to identify individuals at risk of hereditary cancer.

Clinical criteria used to be the main diagnostic tool for CPSs (3–5). Nevertheless, the discovery of cancer susceptibility genes (6) and the decreasing costs of DNA sequencing created a pathway for genetic testing implementation in the diagnostic framework. Genetic testing criteria have evolved rapidly in recent years (7–9). Despite this progress, global disparities exist, and access remains a critical concern (10).

Genetic counseling access, genetic testing costs, and lack of epidemiological hereditary cancer data are barriers to cancer predisposition assessments in Brazil and other Latin American countries (11). In Brazil, health insurance coverage for genetic testing was initiated in 2018. Although this coverage does not include all clinical scenarios eligible for genetic testing according to current international guidelines, it was the beginning of genetic testing access, at least for the Brazilian population with health insurance. Unfortunately, only 25% of Brazilians have health insurance, therefore genetic testing is not yet available for most citizens who depend on the public health system (12).

Epidemiological data are paramount to understand demands and opportunities for cost-effective interventions and resource allocation. The present study explores regional epidemiological data from Brazilian patients at risk for hereditary cancer. We also aimed to compare national and international guidelines criteria for germline genetic testing.

## Methods

Individual patient data were retrospectively collected from medical records of patients referred to the Oncogenetics Department at Hospital Sírio-Libanês (Brasília, Federal District, Brazil) for genetic counseling between July 2017 and January 2021. Patients with a personal history of cancer and/or family history of cancer were included in the analysis. A waiver of informed consent was approved by the Institutional Review Board of Hospital Sírio-Libanês.

Data were anonymized by removing all patient identifiers. The collected data included sex, age at cancer diagnosis, cancer type, number of primary cancers, family history of cancer, and germline genetic test results. Family cancer history was obtained through pedigree analysis and/or information from proband’s medical records. Any cancer in first-, second-, or third-degree relatives was considered a positive family history of cancer. The concept of limited family structure proposed by Weitzel et al. (13), was adapted for this study. Limited family structure was defined as fewer than two first- or second-degree relatives surviving past 45 in either lineage, maternal or paternal. Patients with an unknown family history were also classified as having a limited family structure.

Criteria for germline genetic testing were revised according to national and international guidelines: (i) testing criteria published by the Brazilian National Agency of Supplementary Health (ANS); and (ii) NCCN Clinical Practice Guidelines on Oncology: genetic/familial high-risk assessment for breast, ovarian, and pancreatic cancer (version 2.2021) (14) and genetic/familial high-risk assessment for colorectal cancer (version 1.2021) (15). For hereditary diffuse gastric cancer, we used updated clinical practice guidelines proposed by Gullo etal. (16). Information about the commercial laboratory that performed the germline test, the testing methodology, and the number of genes evaluated were also collected. The classification of the variants described in this paper are those reported by the respective laboratories.

### Statistical analyses

Data were tested for normality using the Kolmogorov–Smirnov and Shapiro–Wilk tests. Values are expressed as medians and percentiles for non-normal continuous variables and as means and standard deviations for normal continuous variables. Categorical data are presented as absolute values and percentages and were tested using the Pearson X2 and Fisher exact tests. Quantitative data were compared by applying Student’s t-test to compare the two groups for normally distributed variables and the Mann–Whitney U test for non-normally distributed ones. Statistical significance was set at P ≤0.05. Statistical analyses were performed using SPSS version 21.0 (IBM, NY, USA).

## Results

### General characteristics of the studied population

In total, 1,091 individuals from 985 families were included in this study. Female patients represented 83.6% (912/1091) of the cohort. At the first genetic counseling session, 66.0% (720/1091) of patients had a personal history of cancer. Thirty-one percent of patients (346/1091) were cancer-unaffected, 2.7% were under investigation for a malignant disease, 10.0% had a recent diagnosis of cancer, 13.0% were receiving oncological treatment (radiotherapy or chemotherapy), 34.6% had already completed cancer treatment, and 5% had metastatic disease. Disease status information was unavailable for 35 patients.

Most patients (93.5%, 1020/1091) had one or more family members affected by cancer. Most of these family members had cancer before the age of 50 years (64.0%, 653/1020). Twenty-three patients (2.1%) had a limited family structure or unavailable family history. One hundred and three patients (9.4%) were referred for genetic testing because of a previous identification of a familial germline pathogenic variant in a blood relative.

Of the 82.5% (900/1091) of patients who fulfilled international guidelines for genetic testing, 60.0% (655/900) were eligible for ovarian, breast, and pancreatic cancer genetic testing, 7.1% (78/900) for Lynch syndrome, 1.6% (18/900) for Li-Fraumeni syndrome, 1.4% (15/900) for adenomatous polyposis syndromes, and 2.8% (31/900) for other CPSs. Considering the ANS criteria, 57.5% (627/1091) of patients were eligible for genetic testing.

### Cancer-affected patients

Approximately 62.0% (446/720) of patients with a personal history of cancer were under the age 50. The median age at first cancer diagnosis was 46 years (interquartile range [IQR], 37-55 years).

The cohort included patients with 30 different cancer types (**Supplementary Material 1**). Breast cancer was the most prevalent tumor (52.9%, 466/880), followed by colorectal cancer (8.3%), ovarian cancer (5.9%), thyroid cancer (3.7%), sarcoma (3.4%), renal cancer (3.2%), prostate cancer (2.6%), pancreatic cancer (2.4%), endometrial cancer (2.2%), neuroendocrine tumor (2.2%), melanoma (2.2%), and gastric cancer (2.0%).

Seventeen percent of patients (127/720) had multiple primary cancers. Of those, most patients had two primary cancers (81.0%, 103/127). A higher risk of multiple primary cancers was associated with young-onset cancers; however, this association was not statistically significant (p=0.263).

### Germline testing results

**Figure 1:**
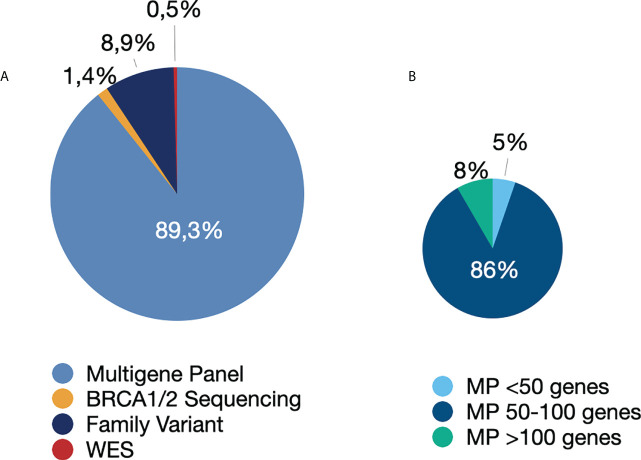
Genetic tests performed. **(A)** Type of genetic test performed. **(B)** Number of genes in the multigene panel. Abbreviations: MP, multigene panel; WES, whole-exome sequencing.

Among the 879 patients who underwent germline genetic testing, 89.3% (n=785) underwent multigene panel testing. The remaining patients were tested using the following strategies: family variant testing (8.9%), BRCA1/BRCA2 gene testing (1.4%), and whole-exome sequencing (0.5%) (**Figure 1**). The four patients who underwent whole-exome sequencing were under investigation for CPSs and other disorders of genetic background, such as hereditary neuropathy, inborn errors of metabolism, and premature ovarian insufficiency.

One hundred and twenty pathogenic or likely pathogenic variants (P/LPVs) in 34 genes were detected in 26.9% (236/879) of the tested patients. According to ACMG’s list of medically actionable genetic findings (17) and/or the list proposed by Desmond et al. (18), the prevalence of actionable P/LPVs would be 24.9% (n=219).

Variants of uncertain significance (VUS) were described in 402 patients (45.7%). **Table 1** summarizes the clinical characteristics of patients who underwent germline testing. Most P/LPVs were found in cancer-affected patients (58.5% vs. 41.5%; p<0.001). Family members affected by cancer under the age of 50 and a history of multiple primary cancers were independently associated with germline P/LPVs identification (p< 0.05).

**Table 1:** Clinical profile of the patients who underwent germline testing for CPSs.

	Positive result N (%)	Negative/VUS resultN (%)	p value
**Personal history of cancer** Yes	138 (58.5)	472 (73.4)	<0.001
No	98 (41.5)	171 (26.6)	
**Age 1st cancer diagnosis** < 18 yrs	1 (0.7)	7 (1.5)	0.456
19- 35 yrs	26 (19.0)	79 (16.8)	
36- 45 yrs	42 (30.7)	137 (29.1)	
46- 49 yrs	12 (8.8)	68 (14.4)	
> 50 yrs	56 (40.9)	180 (38.2)	
Total	137	471	
**N° of primary cancers** 0	98 (41.5)	171 (26.6)	0.039
1	106 (45)	397 (61.7)	
2	25 (10.6)	64 (10)	
3	4 (1.7)	11 (1.7)	
4	2 (0.8)	0	
5	1 (0.4)	0	
Total	236	643	
**Family history of cancer** Yes	224 (94.9)	595 (92.5)	0.349
Negative	7 (3)	34 (5.3)	
Unknown	5 (2.1)	14 (2.2)	
Total	236	643	
**Relatives affected < 50 yrs** Yes	165 (69.9)	367 (57.1)	0.001
No	66 (28)	261 (40.6)	
Unknown	5 (2.1)	15 (2.3)	
Total	236	643	
**Fulfill international criteria** Yes	217 (91.9)	530 (82.4)	<0.001
No	19 (8.1)	113 (17.6)	
Total	236	643	
**Fulfill ANS criteria** Yes	170 (72)	380 (59.1)	<0.001
No	66 (28)	263 (40.9)	
Total	236	643	

VUS, variant of uncertain clinical significance; yrs, years; ANS, Brazilian National Agency of Supplementary Health.

Among 269 cancer-unaffected patients who underwent genetic testing, 36.4% (n=98) harbored a P/LPV. Fifty-four percent (53/98) of those patients were tested due to previous identification of the variant in another family member (cascade testing).

Heterozygous P/LPVs in BRCA2 (18.6%), BRCA1 (14.4%), TP53 (13.6%), MUTYH (9.7%), and CHEK2 (5.9%) were the most frequently reported genetic findings. Fourteen patients harbored a monoallelic P/LPV associated with recessive disorders (NTHL1, RECQL4, ERCC3, FANCA, and BLM). **Table 2** shows the distribution of germline P/LPVs according to the cancer type.

**Table 2:** Distribution of tumors according to germline P/LPVs identification.

Gene (N^o^ of patients harboring germline P/LPVs)																																				
	Number of tumors	P/LPVs (%)	*APC* (4)	*APC (l1307K)* (2)	*ATM* (8)	*AXIN2* (2)	*BARD1* (3)	*BLM* (2)	*BRIP1* (3)	*BRCA1 (34)*	*BRCA2* (44)	*CDH1* (1)	*CHEK2* (14)	*ERCC3* (1)	*FANCA* (1)	*FH* (1)	*MEN1* (2)	*MITF* (4)	*MLH1* (2)	*MSH2* (9)	*MSH6* (1)	*MUTYH mono* (23)	*MUTYH biallelic* (1)	*NF1* (2)	*NTHL1* (7)	*PALB2* (8)	*PMS2* (1)	*PRKAR1A* (1)	*RAD50* (2)	*RAD51C* (9)	*RAD51D* (2)	*RECQL4* (3)	*SDHA* (1)	*SDHB* (4)	*TP53* (32)	*TYR* (2)
			N^o^ of tumors according to germline P/LPVs																																	
Breast	407	23.3%			3	1	4		2	18	21	1	12					1			1	7			2	4			1	2	1	1			12	1
Colorectal	60	26.7%				1		1					3						3	5		1								1	1					
Ovarian	48	33.3%								5	3				1	1				2			1			2				1						
Thyroid	29	20.7%									1		3						1			1														
Sarcoma	24	29.2%									1											1													5	
Renal	17	17.6%											1									1													1	
Prostate	18	33.3%								1	2		1							1											1					
Pancreatic	20	20.0%			1								1												1						1					
Endometrial	16	43.8%								1	2							2				1							1							
NET	13	15.4%															2																			
Melanoma	14	21.4%																				1		1											1	
Gastric	12	8.3%																		1																
Other cancers*	61	32.8%		1	1			1		1	5		1			1								1									1	1	6	
No cancer	-	-	4	1	3	1			1	9	20		8	1				2		3		10			5	2	1	1		5		2		3	15	1

P/LP, pathogenic/likely pathogenic; VUS, variant of uncertain significance; NET, neuroendocrine tumor. *Other cancers included testicular, lymphoma, leukemia, schwannoma, urothelial, bladder, adrenocortical, head and neck, skin (non-melanoma), soft tissue, lung, central nervous system, uterus, parotid, hepatocarcinoma, pheochromocytoma, appendix,gallbladder, and multiple myeloma.Adapted from Samadder et al. (9).Color shading is related to the frequency of each alteration, darker shades represent higher frequencies.

Thirteen patients harbored two P/LPVs. One was homozygous for a pathogenic variant of MUTYH. Four patients (1.7%, 4/236) had an overlap of high/moderate penetrance P/LPVs for autosomal dominant CPSs (**Supplementary Material 2**). Eight patients harbored monoallelic variants in high/moderate penetrance cancer genes associated with autosomal dominant inheritance and a second variant in a gene associated with a recessive disorder (MUTYH, FANCA, NTHL1) or low penetrance cancer (TYR).

Testing criteria

Sixty-two percent of the tested patients (550/879) fulfilled both the international guidelines and ANS criteria, 19.9% fulfilled only the international guidelines criteria, and 8.1% underwent germline testing despite not meeting testing criteria.

Genetic testing criteria from international guidelines were more effective in identifying P/LPV carriers than the ANS criteria (92% vs. 72%, p<0.001). Both approaches would have missed some diagnoses, including approximately 10% (19/191) of patients screened by international guidelines and 14% (66/464) of those screened by ANS criteria.

## Discussion

This study analyzed data of 1,091 Brazilian individuals from 985 different families, referred to genetic counseling due to personal and/or family history of cancer. This is the largest single center Brazilian cohort, from the Center-West of the country, that underwent germline genetic testing with multigene panels for hereditary cancer. Germline genetic tests guide high-risk surveillance, risk-reduction recommendations, and cancer treatment (15, 16, 19–23). The detection rate of germline P/LPVs varies according to criteria selection for testing and testing approaches (24–26). Although most Brazilian patients have limited access to hereditary cancer risk assessment (12, 27), our results provide some insights on genetic testing for hereditary cancer in Brazil.

The present cohort comprised a highly selected population with access to private healthcare and molecular testing. Most patients referred to genetic counseling met clinical criteria for germline testing (82.5%). A personal history of multiple primary cancers and family history of cancer under 50 were important predictors of a positive test result, in line with previous studies (28–30). International criteria more effectively identified P/LPV carriers in cancer susceptibility genes than ANS criteria (92% vs. 72%, p<0.001). Both criteria missed 10-14% of P/LPV carriers. Other studies have already highlighted that current testing criteria may not be able to identify all carriers (31–33). For this reason, some authors advocate for universal screening in some clinical scenarios (9, 34–36). Nevertheless, before advocating for universal genetic testing, we must ensure equitable access to interventions associated with positive test results (37).

In addition, cost-effectiveness of genetic testing may be affected by cascade testing, which involves identifying asymptomatic family members at risk (38). Our study demonstrated that 36.4% of the tested cancer-unaffected patients harbored a P/LPV in a CPS gene. Among these patients, only 54% had a previously identified family variant that made them eligible to pursue genetic testing according to ANS criteria. ANS only endorses germline testing for asymptomatic patients who have a relative with previous identification of a germline P/LPV. This finding should prompt discussion among Brazilian regulatory agencies and medical societies who are involved in revising national guidelines.

Approximately 27% of all tested patients harbored one or more PV/LPVs in cancer susceptibility genes, which is similar to study results from India (39) but higher than other studies (9, 40, 41). We attributed this difference to a highly selected sample including patients with previous identification of a family variant (9%), patients that did not meet genetic testing criteria (8.1%) and the use of large multigene panels (95% ≥ 50 genes). Universal genetic testing is indicated for some cancer types (e.g. epithelial ovarian cancer) and has been debated for other clinical scenarios (e.g. breast cancer, colorectal cancer) (7, 9, 42). Higher rates of positive genetic test results are achieved with the universal testing approach in comparison to the criteria-based (9, 31). In addition, Tsaousis et al. (43) demonstrated that depending on the number of genes included in the multigene panel, the identification of PVs can increase from 15.1% to 24.7%, and the higher range may be attributed to 4.5% of PVs in low-risk/limited data genes. In our cohort, fourteen patients harbored a monoallelic P/LPV associated with recessive disorders (NTHL1, RECQL4, ERCC3, FANCA, and BLM) and one patient harbored PV in a low penetrance cancer gene (TYR). The actionability in carriers of recessive disorders are related to reproductive risks.

The most frequently mutated genes in our cohort were BRCA2 (18.6%), BRCA1 (14.4%), TP53 (13.6%), and monoallelic MUTYH (9.7%). Interestingly, a recently published nationwide Brazilian study, with the largest breast cancer patient cohort (n= 1663) submitted to genetic testing, also described these genes as the most mutated among patients with positive genetic test results (44). In contrast to the cohort from Guindalini’s paper, we included patients with different types of cancer. However, our sample was enriched by breast cancer patients; therefore, a high prevalence of P/LPVs in BRCA1/2 was expected. The high rate of P/LPVs in the TP53 gene described in our study is possibly related to the founder effect that the p.Arg337His (p.R337H) variant exerts in Brazil, and a possible selection bias associated with referrals to our team of specialists in Li-Fraumeni syndrome. This variant is found in up to 0.3% of the population of the southern and southeastern regions of the country (45), and in lower frequencies in other Brazilian regions (46, 47). Unlike BRCA1/2 and TP53, monoallelic MUTYH P/LPVs are not associated with breast cancer but may predict earlier colorectal screening in families affected by colorectal cancer.

Hereditary cancer awareness is growing rapidly. Professional education in hereditary cancer risk assessment, including multidisciplinary team training, strategies to optimize genetic counseling referrals, and genetic testing access, improve CPSs identification rates (11, 48). In Brazil and other low- or middle-income countries, the socioeconomic barrier impact health care access. Despite the worldwide advocacy for broad genetic testing access (49, 50), uninsured patients remain a concern. Providing access to genetic testing without assurance of all subsequent preventive and treatment opportunities may bring more harm than benefit (37). Continuous efforts in private and public settings should be made to pursue equitable hereditary cancer diagnosis and management.

Despite some limitations related to the retrospective nature of this study, as well as, the fact that it consisted of a highly selected sample from a single center, our results might form the basis for prospective studies and national collaborative efforts to achieve higher quality data that will impact policy makers.

## Conclusion

The Brazilian ANS testing criteria should be revised to consider inclusion criteria for germline testing of cancer-unaffected patients with a suspected family history of CPS. Multigene panels provide high rates of P/LPV detection and should be considered a first-tier strategy. Hereditary cancer awareness among health care providers, genetic counseling training, and education for the proper interpretation of genetic test results, including understanding their clinical validity and utility, should be available in private and public settings.

## Data availability statement

The original contributions presented in the study are included in the article/**Supplementary Material**. Further inquiries can be directed to the corresponding author.

## Author contributions

The authors confirm contribution to the paper as follows: study conception and design: AL and RS; data collection: AL and RS; analysis and interpretation of results: AL and RS. Draft manuscript preparation: AL, DS, AP, NM, RB-S, TC, FM, IM, BG, LF, GF, and RS. All authors reviewed the results and approved the final version of the manuscript.

## Conflict of interest

Author DAS participates in clinical studies sponsored by the company Lilly, and as a speaker at events for AstraZeneca, MSD, Novartis, Roche, Pfizer, Advisory, GSK, Roche. Author AALP reported research involvement with MSD; received honoraria from Servier, MERK, MSD, Novartis, Lilly, AstraZeneca; reported logistic support for educational meetings from AmGen/MSD/Roche; and had advisory role from Servier, Bayer, MERK. Author RB-S reported receiving speaker bureau fees from Agilant, AstraZeneca, Daichi-Sankyo, Eli Lilly, Pfizer, Novartis, Merck, and Roche; he has also served as a consultant/advisor for AstraZeneca, Daichi-Sankyo, Eli Lilly, Libbs, Roche, Merck and has received support for attending medical conferences from AstraZeneca, Eli Lilly, Daichi-Sankyo, and Merck. Author TSC participates in clinical studies sponsored by the company Lilly, Pfizer, Novartis, BMS; and reported receiving speaker bureau fees from AstraZeneca, Novartis, Pfizer, Novartis, Libs. Author IAPM is employed as an advisory board by BMS, Bayer, Astellas, Janssen, MSD, ROCHE, AstraZeneca, participates in clinical studies sponsored by the company BMS, Astellas, Janssen; and reported receiving speaker bureau fees from Novartis, Janssen, ROCHE, Astellas, MSD, BMS, IPSEN, AMGEM, AstraZeneca. Author GdSF is employed as an advisory board by BMS, MSD, ROCHE, Astellas, Boeringher, Bayer, and reported receiving speaker bureau fees from BMS, MSD, ROCHE. DAS participates in clinical studies.

The remaining authors declare that the research was conducted in the absence of any commercial or financial relationships that could be construed as a potential conflict of interest.

## Publisher’s note

All claims expressed in this article are solely those of the authors and do not necessarily represent those of their affiliated organizations, or those of the publisher, the editors and the reviewers. Any product that may be evaluated in this article, or claim that may be made by its manufacturer, is not guaranteed or endorsed by the publisher.
