# Supplementary material for: What can we learn from more than 1,000 Brazilian patients at risk of hereditary cancer?
Source: Front Oncol. 2022 Sep 5;12:963910. doi: 10.3389/fonc.2022.963910 (PMC9484549; doi:10.3389/fonc.2022.963910)
Supplement: Supplementary file 1 [file Table_1.docx]

Supplementary material 1. Number of primary tumors according to cancer type.

|  | **Number of primary tumors** | | | |  |
| --- | --- | --- | --- | --- | --- |
| **Cancer subtype** | **1** | **2** | **3** | **4-7** | **Total** |
| Breast | 400 | 57 | 8 | 1 | 466 |
| Colorectal | 62 | 10 | 1 | - | 73 |
| Ovarian | 45 | 6 | 1 | - | 52 |
| Thyroid | 26 | 6 | 1 | - | 33 |
| Sarcoma | 26 | 3 | - | 1 | 30 |
| Gastric | 17 | 1 | - | - | 18 |
| Endometrium | 16 | 3 | - | - | 19 |
| Pancreatic | 16 | 5 | - | - | 21 |
| Prostate | 15 | 7 | 1 | - | 23 |
| Neuroendocrine | 15 | 2 | 2 | - | 19 |
| Renal | 14 | 10 | 3 | 1 | 28 |
| Melanoma | 13 | 6 | - | - | 19 |
| Others | 55 | 12 | 7 | 5* | 79 |
| **Total** | **720** | **128** | **24** | **8** | **880** |

* There was one patient who had 7 basal cell carcinomas and were under investigation of Gorlin syndrome.

Supplementary Material 3. Pathogenic/ likely pathogenic variants

| **Gene** | **Variant** | **Number of patients** |
| --- | --- | --- |
| *APC* | *APC* c.481C>T (p.Gln161*) | 3 |
|  | *APC* c.802G>T (p.Glu268*) | 1 |
|  | *APC* c.3920T>A (p.Ile1307Lys) | 2 |
| *ATM* | *ATM* c.185+1del (splice site) | 1 |
|  | *ATM* c.3682G>T (p.Glu1228*) | 1 |
|  | *ATM* c.4115del (p.Leu1372fs) | 1 |
|  | *ATM* c.5497-2A>G (Splice acceptor) | 1 |
|  | *ATM* c.6975G>A (p.Ala2325Ala)** | 1 |
|  | *ATM* c.7913G>A (p.Trp2638*) | 2 |
|  | *ATM* c.9079dup (p.Ser3027Lysfs*36) | 2 |
| *AXIN* | *AXIN2* c.1966C>T (p.Arg656*) | 1 |
|  | *AXIN2* c.815+1G>C (splice donor) | 1 |
| *BARD1* | *BARD1* c.176_177del (p.Glu59Alafs*8) | 3 |
| *BLM* | *BLM* c.298_299delCA | 1 |
|  | *BLM* Deletion exon 2-4) | 1 |
| *BRCA1* | *BRCA1* c.66dupA (p.Glu23Argfs*18) | 1 |
|  | *BRCA1* c.132C>G (p.Cys44Trp) | 1 |
|  | *BRCA1* c.441+2T>A (splice donor) | 1 |
|  | *BRCA1* c.470_471del (p.Ser157*) | 3 |
|  | *BRCA1* c.791_794del (p.Ser264Metfs*33) | 2 |
|  | *BRCA1* c.850C>T (p.Gln284Ter) | 1 |
|  | *BRCA1* c.1115G>A (p.Trp372*) | 3 |
|  | *BRCA1* c.1687C>T (p.Gln563*) | 2 |
|  | *BRCA1* c.2037delGinsCC (p.Lys679asnfs*4) | 1 |
|  | *BRCA1* c.2157insCC (p.Lys680fs) | 1 |
|  | *BRCA1* c.2256_2260del (p.Ser753Argfs*7) | 1 |
|  | *BRCA1* c.2338C>T (p.Gln789*) | 1 |
|  | *BRCA1* c.2704del (p.Glu902Asnfs*98) | 2 |
|  | *BRCA1* c.3331_3334del (p.Gln1111Asnfs*5) | 2 |
|  | *BRCA1* c.3598C>T p.Gln1200* | 1 |
|  | *BRCA1* c.4214dupT (p.Leu1407Alafs*7) | 1 |
|  | *BRCA1* c.5062_5064delGTT (p.Val1688del) | 2 |
|  | *BRCA1* c.5177_5180delGAAA (p.Arg1726Lysfs*3) | 1 |
|  | *BRCA1* c.5266dup (p.Gln1756Profs*74) | 6 |
|  | *BRCA1* Deletion exon 8-19 | 1 |
| *BRCA2* | *BRCA2* c.2T>G (p.Met1Arg) | 3 |
|  | *BRCA2* c.156_157insAlu | 3 |
|  | *BRCA2* c.1310_1313delAAGA (p.Lys437Ilefs*22) | 1 |
|  | *BRCA2* c.2266C>T (p.Gln756*) | 1 |
|  | *BRCA2* c.2512A>T (p.Lys838*) | 1 |
|  | *BRCA2* c.2806_2809del (p.Ala938fs*) | 1 |
|  | *BRCA2* c.2808_2811 (p.Ala938Profs*21) | 3 |
|  | *BRCA2* c.3680_3681del (p.Leu1227Glnfs*5) | 3 |
|  | *BRCA2* c.3847_3848delGT (p.Val1283Lysfs*2) | 1 |
|  | *BRCA2* c.4005dupA (p.Phe1336Ilefs*2) | 4 |
|  | *BRCA2* c.4829_4830delTG (p.Val1610Glyfs*4) | 2 |
|  | *BRCA2* c.5073dupA (p.Trp1692Metfs*3) | 4 |
|  | *BRCA2* c.5164_5165delAG ((p.Ser1722Tyrfs*4) | 2 |
|  | *BRCA2* c.6405_6409del (p.Asn2135Lysfs*3) | 1 |
|  | *BRCA2* c.6696delA (p.Ala2233Leufs*8) | 2 |
|  | *BRCA2* c.7738C>T (p.Gln2580*) | 1 |
|  | *BRCA2* c.8009C>G (p.Ser2670Trp) | 1 |
|  | *BRCA2* c.9382C>T (p.Arg3128*) | 10 |
| *BRIP1* | *BRIP1* c.2392C>T (p.Arg798*) | 1 |
|  | *BRIP1* c.2990_2993del (p.Thr997Argfs*61) | 2 |
| *CDH1* | *CDH1* c.1686_1687insCG (p.Ala563Argfs*3) | 1 |
| *CHEK2* | *CHEK2* c.319+2T>A (splice donor) | 1 |
|  | *CHEK2* c.349A>G (p.Arg117Gly) | 5 |
|  | *CHEK2* c.470T>C (p.Ile157Thr) | 1 |
|  | *CHEK2* c.478 A>G (p.Arg160Gly) | 3 |
|  | *CHEK2* c.593-1G>T (splice acceptor) | 6 |
|  | *CHEK2* c.846+1G (splice donor) | 3 |
|  | *CHEK2* c.975+1G>C (splice donor) | 1 |
|  | *CHEK2* c.1008+2T>G (splice donor) | 1 |
|  | *CHEK2* Gain (Exons 6-7) | 1 |
| *ERCC3* | *ERCC3* c.325C>T (p.Arg109*) | 1 |
| *FANCA* | *FANCA* c.3788_3790del (p.Phe1263del) | 1 |
| *FH* | *FH* c.1431_1433dupAAA (p.Lys477dup) | 1 |
| *MEN1* | *MEN1* c.1050-2A>G (splice acceptor) | 1 |
|  | *MEN1* c.1252 G>C (p.Asp418His) | 1 |
| *MITF* | *MITF* c.952G>A (p.Glu318Lys) | 4 |
| *MLH1* | *MLH1* c.497_498delinsC (p.Leu166Serfs*36) | 1 |
|  | *MLH1* c.1975C>T (p.Arg659*) | 1 |
| *MSH2* | *MSH2* c.28C>T (p.Gln10*) | 1 |
|  | *MSH2* c.942+3 A>T (Intronic) | 3 |
|  | *MSH6* c.1519dupA (p.Arg507Lysfs*8) | 1 |
|  | *MSH2* c.1959delT (p.Asn653Lysfs*32) | 1 |
|  | *MSH2* c.2152C>T (p.Gln718*) | 3 |
|  | *MSH2* del exons 1-6 e EPCAM del exons 2-6 | 1 |
| *MSH6* | *MSH6* c.1519dupA (p.Arg507Lysfs*8) | 1 |
| *MUTYH* | *MUTYH* c.347-1G>C (p.?) | 1 |
|  | *MUTYH* c.389-1G>C (Splice acceptor) | 2 |
|  | *MUTYH* c.494A>G (p.Tyr165Cys) | 3 |
|  | *MUTYH* c.536 A>G (p.Tyr179Cys) | 4 |
|  | *MUTYH* c.933+3A (Intronic) | 1 |
|  | *MUTYH* c.1145G>A (p.Gly382Asp) | 2 |
|  | *MUTYH* c.1147del (p.Ala385Profs*23) | 4 |
|  | *MUTYH* c.1187G>A (p.Gly396Asp) | 9 |
| *NF1* | *NF1* c.479G>C (p.Arg160Thr) | 1 |
|  | *NF1* c.3113+2_3113+4del | 1 |
| *NTHL1* | *NTHL1* c.268C>T (p.Gln90*) | 6 |
|  | *NTHL1* c.859C>T (p.Gln287*) | 1 |
| *PALB2* | *PALB2* c.93dup (p.Leu32Thrfs*11) | 1 |
|  | *PALB2* c.1633G>T (p.Glu545*) | 1 |
|  | *PALB2* c.1675C>T (p.Gln559*) | 1 |
|  | *PALB2* c.3426dupA (p.Leu1143Thrfs*14) | 1 |
|  | *PALB2* Deletion exon 1-10 | 1 |
|  | *PALB2* Deletion exon 10 | 2 |
|  | *PALB2* Deletion exon 2-3 | 1 |
| *PMS2* | *PMS2* c.631C>T p.(Arg211*) | 1 |
| *PRKAR1A* | *PRKAR1A* c.547delG (p.Asp183Metfs*23) | 1 |
| *RAD50* | *RAD50* c.2467C>T (p.Arg823*) | 1 |
|  | *RAD50* c.2944A>T (p.Lys982*) | 1 |
| *RAD51C* | *RAD51C* c.656T>C (p.Leu219Ser) | 1 |
|  | *RAD51C* c.709C>T (p.Arg237*) | 5 |
|  | *RAD51C* Deletion exon 4 | 3 |
| *RAD51D* | *RAD51D* c.694C>T (p.Arg232*) | 2 |
| *RECQL4* | *RECQL4* c.1166_1167del (p.Cys389Phefs*33) | 2 |
|  | *RECQL4 c.1568_1573delinsCCCCC (p.Ser523fs)* | 1 |
| *SDHA* | *SDHA* c.150+1G>A (Splice donor) | 1 |
| *SDHB* | *SDHB* c.591delC (p.Ser198fs*22) | 2 |
|  | *SDHB* Deletion exon 1 | 2 |
| *SMARCE1* | *SMARCE1* c.871C>T (p.Gln291*) | 1 |
| *TP53* | *TP53* c.1010G>A (p.Arg337His) | 29 |
|  | *TP53* c.733G>A (p.Gly245Ser) | 1 |
|  | *TP53* c.853_856del (p.Glu285Lysfs*59) | 1 |
|  | *TP53* Partial deletion exon 5 | 1 |
| *TYR* | *TYR* c.1217C>T (p.Pro406Leu) | 1 |
|  |  |  |

** This variant had conflicting classifications of pathogenicity

Suplementary material 3. Clinical description of patients that harbored an overlap of autosomal dominant CPSs.

| ID | Variant description | Additional Variant description | Gender | Personal history of cancer | Age at cancer diagnosis | Family history of cancer |
| --- | --- | --- | --- | --- | --- | --- |
| BSB0028001 | *BRCA1* c.4214dupT (p.Leu1407Alafs*7) | *MSH2* c.28C>T (p.Gln10*) | Female | Breast | 47 | Maternal uncle- lung cancer at 60 yrs (smoker), maternal aunt- lung cancer > 65 yrs (smoker), paternal uncle- unknown cancer at 65 yrs, paternal cousin- breast cancer at 41 yrs, paternal grandmother breast cancer > 50 yrs. |
| BSB0166002 | *TP53* c.1010G>A  (p.Arg337His) | *BRCA2* c.4829_4830delTG (p.Val1610Glyfs*4) | Female | No | N/A | Mother (TP53  c.1010G>A carrier)- 5 primary tumors: schwannoma at 39 yrs, melanoma at 50 yrs, squamous cell carcinoma of the neck at 50 yrs, retroperitoneal leiomyosarcoma at 56 yrs, breast cancer at 59 yrs. Paternal uncles with unspecified cancers of the gastrointestinal tract > 50 yrs. |
| BSB0972001 | *BRIP1* c.2990_2993del (p.Thr997Argfs*61) | *BRCA2* c.8009C>G (p.Ser2670Trp) | Female | Breast | 53 | Sister- breast cancer at 44 yrs, another sister- breast cancer at 34 yrs, father- prostate cancer at 78 yrs and pancreatic cancer at 84 yrs, 2 paternal aunts had breast cancer >50 yrs. |
| BSB0311001 | *PALB2* c.93dup (p.Leu32Thrfs*11) | *ATM* c.185+1del (splice site) | Female | Breast | 54 | Father- prostate cancer at 70 yrs, maternal uncle- leukemia at 70 yrs, maternal grandfather- liver cancer at 70 yrs. |

Abbreviations: N/A, not applicable; yrs, years.
